# Supplementary material for: Lizard Blastema Organoid Model Recapitulates Regenerated Tail Chondrogenesis
Source: J Dev Biol. 2022 Feb 10;10(1):12. doi: 10.3390/jdb10010012 (PMC8883911; doi:10.3390/jdb10010012)
Supplement: Supplementary file 1 [file jdb-10-00012-s001.zip › jdb-1514399-supplementary.pdf]

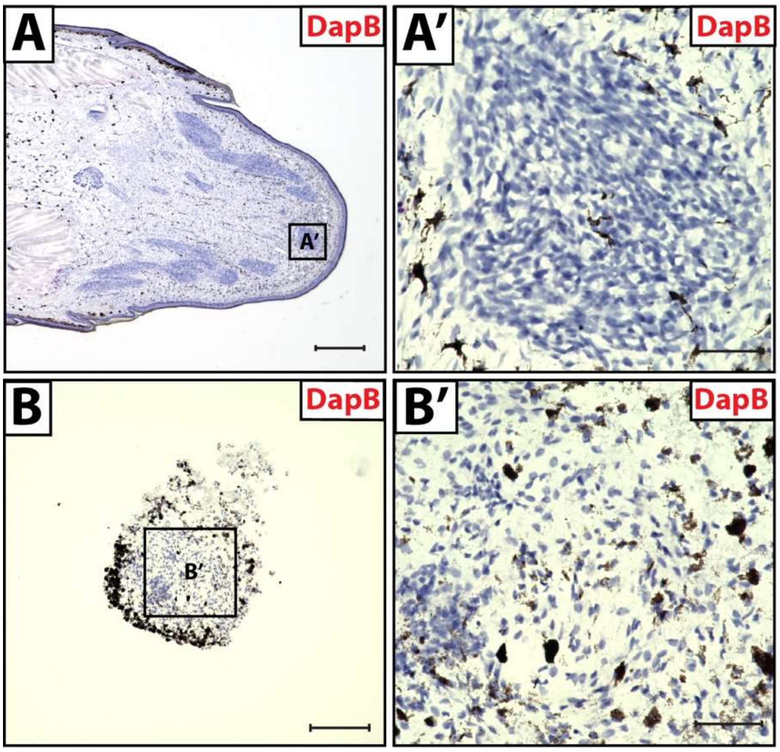

Figure S1. RNAscope negative control in lizard blastema and cell pellets. (**A**) Control lizard tail blastema (D14) analyzed by in situ hybridization for bacterial DapB expression. (**A′**) Higher magnification view of blastema cell region identified in Panel A demonstrating lack of DapB expression in blastema cells. (**B**) Control cell pellet isolated via Trypsin protocol cultured in mammalian media analyzed by in situ hybridization for bacterial DapB expression. (**B′**) Higher magnification views of cell pellet identified in Panel B demonstrating lack of DapB expression in cultured cells. (**A**) Scale bar = 500 μM. (**B**) Scale bar = 200 μM. (**A′**,**B′**) Scale bar = 50 μM.
